# Blending controlled-release urea and urea under ridge-furrow with plastic film mulching improves yield while mitigating carbon footprint in rainfed potato

**DOI:** 10.1038/s41598-022-25845-4

**Published:** 2023-03-10

**Authors:** Mengyuan Sun, Bin Ma, Peina Lu, Jianhui Bai, Junzhen Mi, Jinghui Liu

**Affiliations:** 1 National Outstanding Agriculture Research Talents Innovation Team, Inner Mongolia Agricultural University, Hohhot, Inner Mongolia 010019 People’s Republic of China; 2 Inner Mongolia Academy of Agricultural &Animal Husbandry Science, Hohhot, 010010 People’s Republic of China; 3 Institute of Forestry and Grassland Ecology, Ningxia Academy of Agriculture and Forestry Sciences, Yinchuan, Ningxia 750002 People’s Republic of China; 4 State Key Laboratory of Aridland Crop Science, Gansu Agricultural University, Lanzhou, Gansu, 730070 People’s Republic of China

**Keywords:** Plant sciences, Environmental sciences

## Abstract

Ridge-furrow with plastic film mulching and various urea types have been applied in rainfed agriculture, but their interactive effects on potato (*Solanum tuberosum* L.) yield and especially environments remain poorly understood. A three-year experiment was conducted to explore the responses of tuber yield, methane (CH_4_) and nitrous oxide (N_2_O) emissions, net global warming potential (NGWP), carbon footprint (CF), and net ecosystem economic budget (NEEB) of rainfed potato to two mulching practices [plastic film mulching (RM) and no plastic film mulching (NM)] and three urea types [conventional urea (U), controlled-release urea (C), and a mixture of equal amounts of conventional urea and controlled-release urea at a ratio of 1:1 (CU)] and their interactions. The results showed that RM significantly decreased cumulative N_2_O emissions and CH_4_ uptake by 4.9% and 28.4%, but significantly increased NGWP by 8.9% relative to NM. Compared with U, the C and CU produced much lower cumulative N_2_O emissions and NGWP and higher CH_4_ uptake. The interaction of mulching methods and urea type had significant influence on tuber yield and NEEB. Considering both environment and production, RMCU could not only achieve a high tuber yield and NEEB (by up to 26.5% and 42.9%, respectively), but also reduce the CF (by up to 13.7%), and therefore should be considered an effective strategy for dryland potato.

## Introduction

Global warming, caused primarily by a remarkable increase in the atmospheric concentrations of greenhouse gases (GHG), namely methane (CH_4_) and nitrous oxide (N_2_O), and food security problems, have become global issues^1,2^. Two of the most significant GHG are CH_4_ and N_2_O, which have a strong infrared absorption capacity and large warming effects in the atmosphere. In addition, the global warming potential (GWP) of CH_4_ and N_2_O are 34 and 298 times greater than carbon dioxide (CO_2_) over a 100-year timeframe, respectively^3^. In particular, agriculture soils have been estimated to contribute 84% and 52% to the global anthropogenic N_2_O and CH_4_ emissions, respectively^4,5^. More than 70% of the arable land in north and northwest China is used for rainfed agriculture, mainly located in arid and semiarid areas^6^. Potato (*Solanum tuberosum* L.) is one of the main crops in dry farmland of China^7^, but crop production is limited by water shortage and suboptimal nutrient management^8^. Thus, there is an urgent need to determine optimal strategies to improve crop production while minimizing environmental costs^9,10^.

Ridge-furrow with plastic film mulching system (RM) is a low-cost micro-field rainwater-collecting technique, which has been widely applied to crop production, especially in the arid and semiarid areas^11,12^. Previous studies found that RM increased tuber yield, water productivity, and nitrogen (N) use efficiency^13,14^, by increasing soil temperature and moisture, and improving the availability of soil nutrients in dry farmland^15^. Plastic film mulching has a complex effect on the production, consumption, and transport of CH_4_ and N_2_O in soils by altering the hydrothermal conditions^16,17^. In particular, Yu et al.^17^ found that plastic film mulching significantly reduced CH_4_ emissions from paddy fields by 64.2% and CH_4_ uptake in uplands by 16.1%; and increased N_2_O emissions by 23.9%. However, until now, studies on GHG emissions under plastic film mulching showed inconsistent results in agricultural fields^17^. It has been reported that plastic film mulching significantly increased GHG emissions, greenhouse gas intensity (GHGI), and net global warming potential (NGWP)^18,19^, but the opposite results still exist^20–22^. The contradiction may be caused by inconsistent investigations of agronomical measures, as well as the corresponding soil texture and meteorological characteristics^23^.

N fertilizers have been identified as a viable means to increase crop yield by 30–50%, and more than half the world’s population depends on N fertilizers for food production^23–25^. Urea is the most widely used N fertilizer globally because of its high N content, favorable cost, and ease of application^26,27^. When applied to soil, urea quickly decomposes under the influence of precipitation, and it is easy to cause soil N losses through various pathways such as N_2_O emissions, ammonia volatilization, and leaching, which cannot meet the crop demand for N at the later growth stages^28,29^. Developing high-efficiency fertilizers, such as controlled-release urea (CRU) is thus a promising way to address these issues. CRU has been widely used to improve synchrony between soil N availability and crop N demand, thereby increasing crop yield and mitigating N_2_O emissions^23,30,31^. A meta-analysis by Zhang et al.^23^ indicated that CRU can increase maize yield (5.3%) and N use efficiency (24.1%) and reduce N_2_O emissions (23.8%), compared with urea. However, a single CRU is generally expensive, and the N release rate can be affected by soil moisture and temperature, which may inhibit crop growth at the early growth stage^32–34^. In view of this, more and more researchers suggested that a better management strategy for mixed CRU and urea (CU) has been considered as a better alternative N management strategy to ensure N supply at the early stages and reduce fertilizer costs, thereby increasing crop yield and economic benefit^29,35,36^. Previous study showed that the 1:1 ratio of CRU and common urea could not only achieve a high grain yield, but also enhance ecosystem economic benefit^37^. In light of China’s goal to implement the plan for zero growth of fertilizer, CU offers an excellent option for increasing N use efficiency while minimizing environmental pollution^32,38^. For example, Bai et al.^39^ observed that CU not only improved the economic efficiency of maize cultivation (increased by 4.9–12.1%) but also significantly decreased the NGWP and carbon footprint (CF) (decreased by 21.1–21.7% and 17.8–23.1%, respectively), but net ecosystem economic budget (NEEB) was not assessed.

Previous studies have not investigated the interactive effects of RM with various urea types for potato production on CF and NEEB in rainfed agriculture. Therefore, a comprehensive analysis of the environmental and agronomic effects of RM combined with various urea types are essential. We hypothesized that the combination of RM and various urea types could better balance the production and environmental benefits of rainfed potato in comparison with both strategies individually. This study was aimed to: (1) investigate the dynamics of CH_4_ and N_2_O emissions in a rainfed potato field under RM and various urea types; (2) determine the optimal combination of RM and urea types to increase tuber yield and NEEB, and reduce NGWP and CF; and (3) propose an appropriate combination of RM and urea type for sustainable potato production in dryland areas.

## Materials and methods

### Site description

A three-year (2018–2020) field experiment was conducted at the experimental farm of Qingshuihe County Research Centre (39°57′N, 111°39′E), Hohhot, Inner Mongolia, in north-central China. The preceding crop for each trial was maize (*Zea mays* L.), the most common potato rotational crop in Inner Mongolia. This region has a typical semi-arid mid-temperate continental monsoon climate in a loess hilly-gully region on the Loess Plateau of China. The site has an elevation of ~ 1374 m with an annual mean air temperature of 7.1 °C, total rainfall of 365 mm, and potential evaporation of 2577 mm^40^. The field has a sandy loam texture, with soil fractions of 728 g kg^−1^ sand, 134 g kg^−1^ silt and138 g kg^−1^ clay content^40^. The field experiment soil properties within 20- cm are shown in Table 1.

**Table 1:** The physical chemical properties at 0–20 cm depth at the experimental field.

Soil physicochemical	2018	2019	2020
Soil water content (%)	7.0a	6.8a	2.4b
Available potassium (mg kg^−1^)	103.1a	80.3c	96.2b
Available phosphorus (mg kg^−1^)	7.1a	4.9c	5.8b
Organic matter (g kg^−1^)	9.8a	4.9c	6.6b
Total nitrogen (g kg^−1^)	0.8a	0.4c	0.5b
Total carbon (g kg^−1^)	10.9a	8.7c	9.7b
TC/TN	13.4c	19.8a	18.3b

Numbers followed by different letters in each row indicate significantly differences at α = 0.05 based on ANOVA test.

Rainfall and average monthly temperature during the potato growing seasons was shown in Table 2. The highest average monthly temperature and precipitation of the past 37 years were recorded in July and August, respectively (1981–2017). From May to September, total precipitation was 354.4, 300, and 268.9 mm in 2018, 2019, and 2020, and the average air temperature was 19.9, 19.1, and 19.5 °C, respectively.

**Table 2:** The rainfall and monthly mean air temperature at the experimental site in 2018–2020 growing seasons.

Month	Rainfall (mm)				Temperature (°C)			
	2018	2019	2020	LTA	2018	2019	2020	LTA
May	71.4	29.8	3.0	29.5	17.7	15.7	17.3	16.8
Jun	7.1	49.9	36.0	56.7	22.1	21.9	22.3	20.7
Jul	160.1	97.2	105.7	105.6	23.5	22.1	22.3	23.0
Aug	84.3	66.1	81.2	85.6	23.3	19.5	21.0	20.9
Sep	31.5	57.0	43.0	59.5	13.0	16.3	14.9	15.2
May-Sep	354.4	300	268.9	336.9	–	–	–	–

LTA the average value for the last 37 years (from 1981 to 2017).

### Experimental design

**Figure 1:**
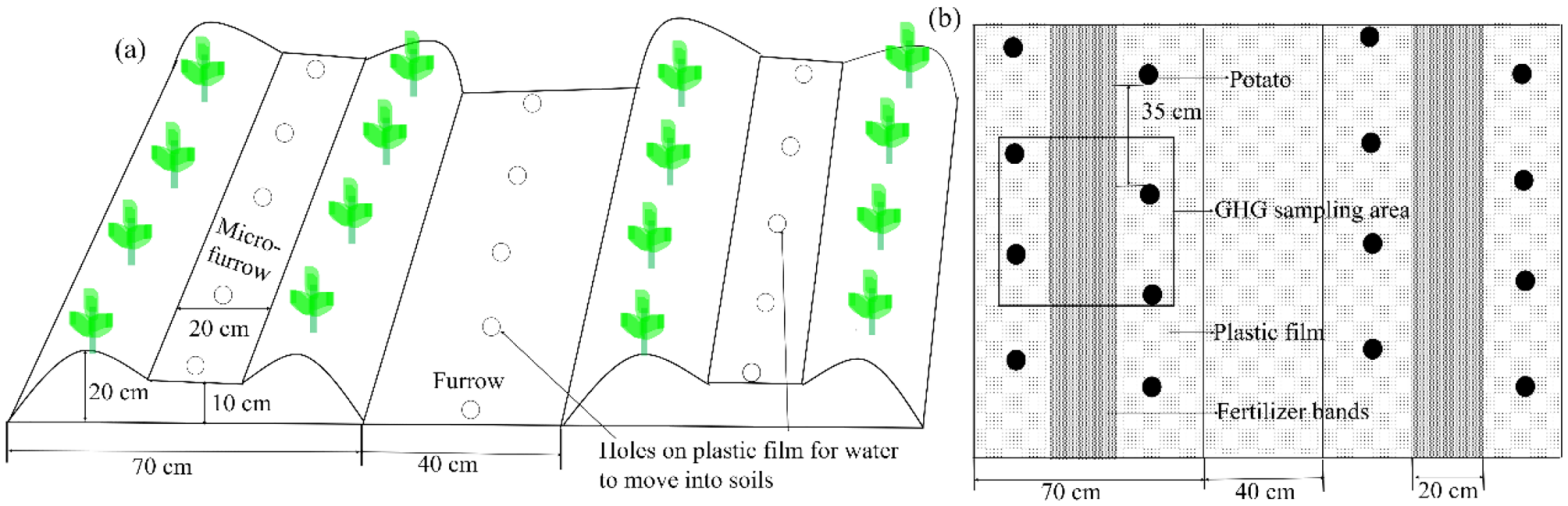
Experimental layout for ridge-furrow with plastic film mulching cultivation systems.

The experiment was a randomized split-plot design with two mulch practices—plastic film mulching (RM) and no plastic film mulching (NM)—as main plots, and three urea types (conventional urea (U), controlled-release urea (C), and a mixture of equal amount of conventional urea and controlled-release urea at 1:1 ratio (CU)) as split plots. There were six treatments: RMU, RMC, RMCU, NMU (local conventional agricultural management), NMC, and NMCU. Each plot was replicated three times. Each plot was 7 m × 7 m, and was surrounded by ridges to prevent surface runoff. Ridge-furrow with plastic film mulching system were used to cultivate potato (Fig. 1a). The width of the ridges base was 70 cm, while the height of the ridges was 20 cm. The furrow width was 40 cm. The width and depth of the micro-ditch within the ridge were 20 cm and 10 cm, respectively.

### Plant materials and agronomic practices

We acknowledge the use of plant materials in this manuscript complies with all relevant institutional, national, and international guidelines and legislation. The potato (‘Kexin 1’) is widely cultivated in dryland regions of China. All seed tubers were obtained from Inner Mongolia Zhengfeng Seed Potato Company, Hohhot of China. Potato was planted on May 13th, 2018, May 20th, 2019, and May 22th, 2020, and harvested on September 18th, 2018, September 23th, 2019, and September 28th, 2020, respectively. Two rows of potato were planted in the ridges with a plant spacing of 35 cm, resulting in a planting density of 52,000 plants ha^−1^. The plastic film was white polyethylene film with 120 cm wide and 0.01 mm in thickness. All fertilizers were applied as basal fertilization in the middle of the ridges at 15 cm soil depth before planting at a rate of 180-120-150 (N-P_2_O_5_-K_2_O) kg ha^−1^. The N fertilizer was in the form of conventional urea (46% N) or controlled-release urea (44.5% N). The latter was a polyurethane-coated urea granules with a nutrient release cycle of approximately 90 days (VIKO Global Technology Ltd, Ningxia, China). The controlled-release urea is coated with nano resin technology; the coating micropore diameter is only a few hundred nanometers, which is an ecofriendly material in the production process with good nutrient release properties. A series of holes were pierced through the surface of the plastic films by iron wires at an interval of 30 cm in each plot. All plastic films were removed by farmers after harvest. No irrigation was performed through the three growing seasons.

### Gas sampling and analysis

A static closed-chamber method was used to estimate the GHG (CH_4_ and N_2_O) emissions for the potato growing seasons^41^. The static closed-chamber consisted of a stainless-steel top chamber (50 × 50 × 60 cm) and a stainless-steel base frame with a groove (50 × 50 × 15 cm) (Fig. S1). Each side of the top chamber was covered with Styrofoam thermal insulation to prevent the air temperature within the chamber from sharply rising during the sampling period in the summer. In addition, the stainless-steel top chamber was equipped with a digital thermometer on the outside, and two small electric fans were installed at the top of each chamber in opposite locations to ensure complete air mixing. There was equipped with a sampling gas channel with a three-way stopcock on the side of the chamber to connect the syringe. After planting the potato, the stainless-steel base frame was inserted at a depth of 12 cm into the soil on the ridge so that the troughs were parallel to the surface and did not move throughout the growing season (Fig. 1b). Chambers were maintained in the open state, except for gas sampling time in the field throughout the potato cultivation period.

Gas samples were collected five times at 1, 3, 5, 7, and 15 days after potato sowing, then about every 15 days thereafter in 2018 and 2019, while at 7 days intervals in 2020, excluding the fallow season. At each measurement, the chambers were placed into the base frame groove filled with water, and then four gas samples were collected for each flux measurement at 10 min intervals from 8:30 to 11:30 am using a 50 mL plastic syringe. The temperature in the chamber was also recorded during each gas extraction. The collected gases were stored in 300 mL gas bags, and the gas samples were taken back to the laboratory for determination of N_2_O and CH_4_ content by PicarroG2308N_2_OCH_4_H_2_O analyzer from Picarro, USA.

Gas emission fluxes were estimated using the increased gas concentration per unit chamber area for a specific time interval^19^:

1 $$\mathrm{F}=\uprho \times \mathrm{H}\times 273/(273+\mathrm{T})\times \mathrm{dc}/\mathrm{dt}$$

where F is the flux of the (mg m^−2^ h^−1^ for N_2_O and CH_4_); dc/dt is the difference of gas concentration inner the chamber headspace; H is the chamber height (m); ρ is the density of each gas at the standard condition (kg m^−3^); T is chamber temperature (°C).

The cumulative GHG emissions for the entire growing season were computed using the following equation:

2 $$\mathrm{G}=( {\sum }_{\mathrm{i}=1}^{\mathrm{n}}\frac{{\mathrm{F}}_{\mathrm{i}}\times {\mathrm{d}}_{\mathrm{i}}}{\mathrm{d}} \times 24\times \mathrm{d})/100$$

where G (kg ha^−1^) is the cumulative emissions of N_2_O and CH_4_; ${F}_{i}$ is the GHG emission flux at the *i* sampling; ${d}_{i}$ is the number of days between the *i* sampling and the next sampling; d is the total number of days in the growth period; 100 is the unit conversion coefficient.

The net global warming potential (NGWP) is commonly used to estimate the relative potential for GHG emissions from agricultural practices^39^. CO_2_-eq emissions are generated by crop production processes, including the production and transportation of plastic film mulch, N, P, and K fertilizers, and herbicides, as well as from diesel used in farm operations. The NGWP equations employed were as follows^39^:

3 $$\sum {\text{GWP}}\;\left( {{\text{others}}} \right) = {\text{EF}}_{{{\text{Herbicide}}\;{\text{rate}}}} \times {\text{Herbicide}}\;{\text{rate}}\;\left( {{\text{kg}}\;{\text{ha}}^{ - 1} } \right) + {\text{EF}}_{{{\text{N}}\;{\text{rate}}}} \times {\text{N}}\;{\text{rate}} + {\text{EF}}_{{{\text{P}}_{2} {\text{O}}_{5} \;{\text{rate}}}} \times {\text{P}}_{2} {\text{O}}_{5} \;{\text{rate}}\;({\text{kg}}\;{\text{ha}}^{ - 1} ) + {\text{EF}}_{{{\text{K}}_{2} {\text{O}}\;{\text{rate}}}} \times {\text{K}}_{2} {\text{O}}\;{\text{rate}}\;({\text{kg}}\;{\text{ha}}^{ - 1} ) + {\text{EF}}_{{{\text{Plastic}}\;{\text{film}}}} \times {\text{Plastic}}\;{\text{film}}\;\left( {{\text{kg}}\;{\text{ha}}^{ - 1} } \right) + {\text{EF}}_{{{\text{Diesel}}\;{\text{fuel}}}} \times {\text{Diesel}}\;{\text{fuel}}\;\left( {{\text{kg}}\;{\text{ha}}^{ - 1} } \right)$$

where the estimated average coefficient factors of EF_Herbicide rate_, EF_N rate_, ${\text{EF}}_{{{\text{P}}_{2} {\text{O}}_{5} \;{\text{rate}}}}$, ${\text{EP}}_{{{\text{K}}_{2} {\text{O}}\;{\text{rate}}}}$, EF_Plastic film_, and EF_Diesel fuel_ were 8.3^39^, 0.79^42^, 0.55^42^, 2.80^43^, 3.75^42^ and 10.15^44^, respectively.

4 $${\text{NGWP}} = {\text{G}}\left( {{\text{N}}_{{2}} {\text{O}}} \right) \times {298} + {\text{G}}\left( {{\text{CH}}_{{4}} } \right) \times {34} + \sum {\text{GWP}}\;\left( {{\text{others}}} \right) + \Delta {\text{SOC}}$$

where NGWP is the net global warming potential (kg CO_2_-eq ha^−1^); G (N_2_O) and G (CH_4_) were the cumulative CH4 emissions and cumulative N_2_O emissions, respectively. Factors 298 and 34 are the default GWP of N_2_O and CH_4_, respectively, for a 100-year time horizon^4,5^. Due to the short duration of this experiment, ∆SOC was neglected.

The carbon footprint (CF) is expressed as the intensity of greenhouse gases produced per unit of production and was calculated using the following equation:

5 $${\text{CF}}\;\left( {{\text{kg}}\;{\text{CO}}_{{2}}{-}{\text{eq}}\;{\text{t}}^{{ - {1}}} } \right) = {\text{NGWP}}/{\text{Yield}}$$

### Soil water content and temperature

At the same time as the gas samples were collected, three soil samples (0 − 20 cm) were randomly collected from the areas between two plants in the ridges using a 5-cm diameter soil auger. Next, three samples were combined to obtain a single aggregated sample for each plot, which was dried at 105 °C for soil gravimetric water content. An automatic temperature recorder (Fotel L93-4 Thermal Instruments, Shanghai China) was buried between two plants in the ridges of each plot and used to record hourly soil temperature at 10 cm soil depth during the potato growing seasons.

### Tuber yield and net ecosystem economic budget

At harvest, 10 plants were randomly sampled from each plot and all the tubers were weighed and recorded to determine yield. The total tubers were weighed for yields and were divided into two classes: commercial tuber > 150 g per tuber and small tuber < 150 g per tuber.

Potatoes with a tuber fresh weight > 150 g were sold at a price of ~ 0.25 USD kg^−1^ and tuber fresh weight < 150 g was sold at a price of ~ 0.16 USD kg^−1^. The price of urea and CRU was estimated at 0.28 USD kg^−1^ and 0.5 USD kg^−1^, respectively. The costs of agricultural operations (sowing + harvest + herbicide), plastic film mulching, fertilizer (P and K fertilizer), and seeds were 634.2, 369.0, 199.7, and 1268.5 USD ha^−1^, respectively. CO_2_-eq was sold at a price of ~ 16.2 USD t^−1^.

The net ecosystem economic budget (NEEB) is an important reference index for crop production and agricultural activities. The NEEB (USD ha^−1^) was calculated using the following equation^45^:

6 $${\text{NEEB}} = {\text{Yield}} \times {\text{Tuber price}} - {\text{Input}} - {\text{NGWP}}\;{\text{cost}}$$

7 $${\text{Input}} = {\text{Costs}}\;{\text{of}}\;{\text{sowing}}\,+\,{\text{herbicide}}\,+\,{\text{plastic}}\;{\text{film}}\;{\text{mulching}}\,+\,{\text{fertilizer}}\,\left( {{\text{N}},\;{\text{P}}\;{\text{and}}\;{\text{K}}} \right)\,+\,{\text{seeds}}\,+\,{\text{harvest}}$$

8 $${\text{NGWP}}\;{\text{cost}} = {\text{NGWP}} \times {\text{CO}}_{2}{-}{\text{eq}}\;{\text{price}}$$

### Statistical analysis

Statistical analyses were carried out in the R environment (version 4.1.1), ANOVA (PERMANOVA) with the “lme4” and “lmerTest” packages to evaluate the influence of different treatments on the tuber yield, NEEB, GHG emissions, NGWP, and CF. Differences were considered statistically significant when *p* ≤ 0.05. The correlation between gas emissions and soil water content and temperature was performed using the Pearson correlation analysis. Figures were drawn using the Origin 2021 software (Origin Lab, USA, http://www.originlab.com/).

## Results

### Soil water content and temperature

Topsoil (0–20 cm) water content for various treatments during the potato growth stage was graphed in Fig. S2. The soil water content varied from 4.4% to 12.2% in 2018, 3.3% to 9.2% in 2019, and 2.6% to 9.6% in 2020. On average, soil water content was 8.0%, 6.6%, and 7.0% under RM, 7.3%, 6.0%, and 6.2% under NM in 2018, 2019, and 2020, respectively. The soil water content was higher under RM than that under NM during the growing seasons. The average temperature for different treatments during the potato growth period was shown in Fig. S2. The soil temperature ranged from 15.4 to 27.1 °C, with an average of 22.4 °C in 2018, from 16.1 to 27.7 °C, with an average of 22.7 °C in 2019, and from 18.4 to 29.3 °C, with an average of 23.4 °C in 2020. On average, soil temperature was 23.2, 23.7, and 24.2 °C under RM, 21.5, 21.8, and 22.7 °C under NM in 2018, 2019, and 2020, respectively. RM increased soil temperature by an average of 1.6–1.9 °C compared with NM over the three growing seasons.

### Soil CH_4_ uptake

**Figure 2:**
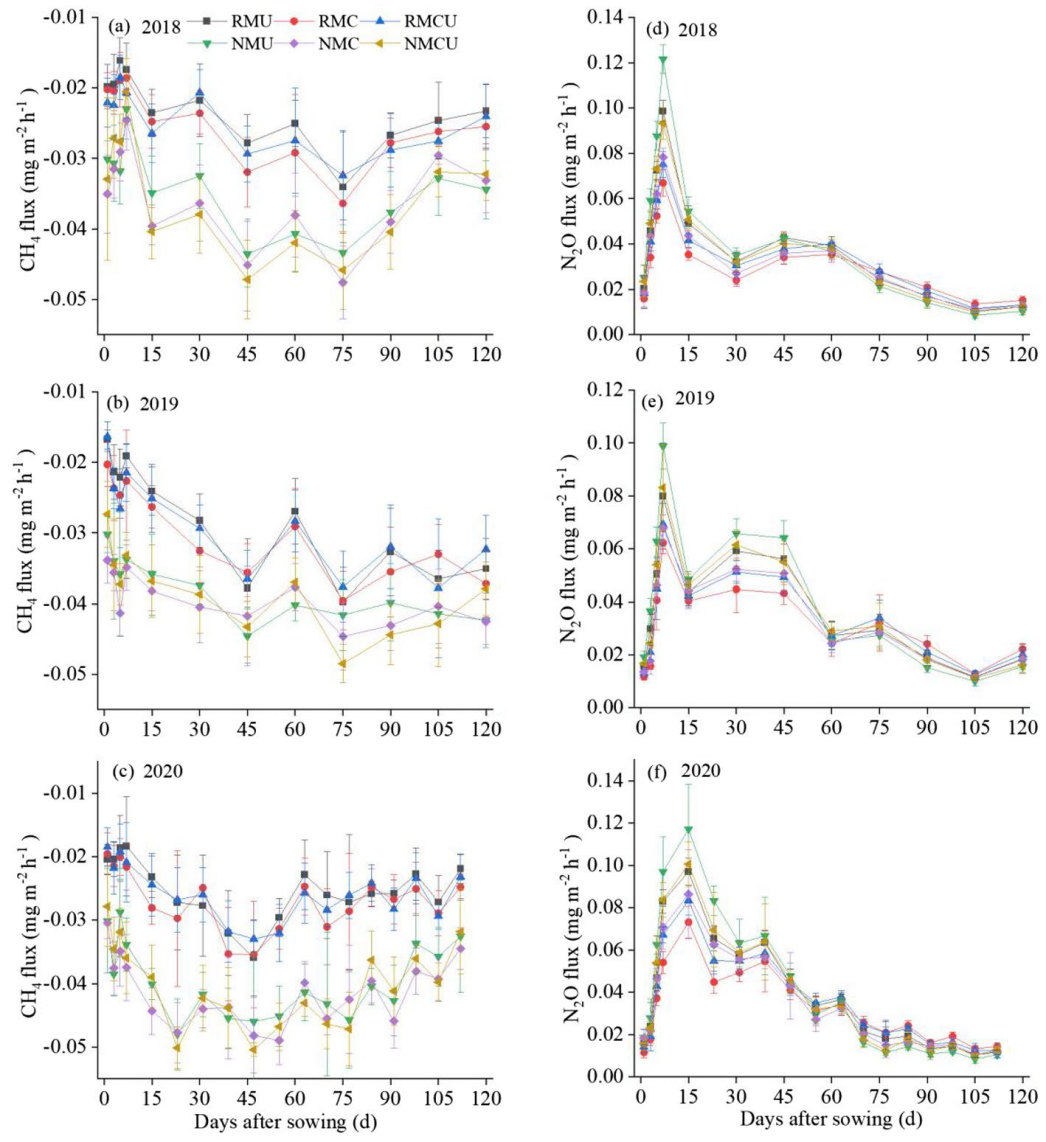
Dynamics of soil CH_4_ fluxes (**a**–**c**) and N_2_O fluxes (**d**–**f**) under different treatments in 2018–2020 growing seasons. *RM* plastic film mulching, *NM* no plastic film mulching, *U* conventional urea, *C* controlled-release urea, *CU* a mixture of equal amount of conventional urea and controlled-release urea at 1:1 ratio. The vertical bars represent the standard error, n = 3.

Seasonal variations of soil CH_4_ fluxes were basically consistent in all treatments, but showed an inconspicuous seasonal variation (Fig. 2a–c). Temporal variations of CH_4_ fluxes were negative in various treatments, indicating that the soil absorbed CH_4_ from the atmosphere over the three growing seasons. The CH_4_ fluxes were significantly and positively correlated with the soil temperature, while a significant negative correlation was observed between CH_4_ fluxes and soil water content during the growing seasons (Table S1).

Mulching (M), urea type (T), year (Y), and M × Y significantly affected the cumulative CH_4_ uptake (Table 3). The cumulative CH_4_ uptake varied from 658.3 to 1074.2 g ha^−1^ under various treatments in 2020, which were lower than the corresponding values in 2018 (722.3 to 1112.6 g ha^−1^) and 2019 (882.7 to 1161.5 g ha^−1^) (Table 3). On average, the cumulative CH_4_ uptake was 29.0% lower under RM than under NM. In addition, the cumulative CH_4_ uptake was increased by 4.4% under C and 3.5% under CU in comparison to U, respectively. However, no significant difference was observed among C, U, and CU for the cumulative CH_4_ uptake under the same mulching practices. The RMU, RMC, and RMCU had significantly lower cumulative CH_4_ uptake, with average reductions of 29.4%, 25.2%, and 27.4%, relative to NMU, respectively. Cumulative CH_4_ uptake under NMCU reached the maximum values of 1112.6 g ha^−1^ and 1161.5 g ha^−1^ in 2018 and 2019, while the corresponding values were 1074.2 g ha^−1^ under NMC in 2020.

**Table 3:** Cumulative CH_4_ uptake and N_2_O emissions, Net global warming potential (NGWP) and carbon footprint (CF) under different treatments in 2018–2020 growing seasons.

Year	Treatment	Cumulative CH_4_ uptake (g ha^−1^)	Cumulative N_2_O emissions (g ha^−1^)	NGWP (kg CO_2_-eq ha^−1^)	CF (kg CO_2_-eq t^−1^)
	RMU	722.3b	1011.0ab	2571.2a	87.2a
	RMC	785.3b	853.6e	2522.2b	86.8a
	RMCU	764.1 b	938.7cd	2548.3ab	76.8b
	NMU	1047.3a	1054.8a	2359.0c	85.1a
	NMC	1086.1a	904.6d	2313.0d	85.9a
	NMCU	1112.6a	984.2bc	2335.8cd	80.0b
2019	RMU	882.7b	1071.3ab	2583.8a	121.2b
	RMC	913.7b	941.9c	2544.1b	123.7b
	RMCU	891.5b	1015.6bc	2566.8ab	112.7c
	NMU	1126.2a	1148.7a	2384.3c	129.5ab
	NMC	1152.0a	984.0c	2334.4d	134.1a
	NMCU	1161.5a	1096.8ab	2367.7c	122.6b
2020	RMU	658.3b	1017.2ab	2575.3a	104.1ab
	RMC	701.1b	879.5c	2532.8b	104.7ab
	RMCU	673.2b	962.4bc	2558.4ab	94.0c
	NMU	1034.3a	1085.7a	2368.7c	106.9a
	NMC	1074.2a	930.4bc	2321.0d	108.4a
	NMCU	1058.0a	1012.3ab	2346.0cd	101.2b
**Significance of factor**					
Mulching (M)		***	***	***	***
Urea type (T)		*	***	***	***
Year (Y)		***	***	**	***
M × T		NS	NS	NS	NS
M × Y		***	NS	NS	***
T × Y		NS	NS	NS	NS
M × T × Y		NS	NS	NS	NS

*RM* plastic film mulching, *NM* no plastic film mulching, *U* conventional urea, *C* controlled-release urea, *CU* a mixture of equal amount of conventional urea and controlled-release urea at 1:1 ratio.

Numbers followed by different letters in each column indicate significantly differences at α = 0.05 based on ANOVA test. The statistical significance is denoted by *P ≤ 0.05; **P ≤ 0.01; ***P ≤ 0.001, and *NS* not significant.

### Soil N_2_O emissions

Similar seasonal dynamics of soil N_2_O fluxes were observed in different treatments, which were mainly dependent on fertilization applications during the potato growing seasons and also driven by rainfall events (Fig. 2d–f). The N_2_O fluxes peaked approximately 7 days after fertilization in 2018 and 2019, and peaked about 15 days after fertilization in 2020. The fluxes then gradually declined and maintained a relatively low level except for some small peaks after rainfall. The N_2_O fluxes increased after fertilization application, with maximum peaks of 0.13 mg m^−2^ h^−1^ in 2018, 0.09 mg m^−2^ h^−1^ in 2019, and 0.12 mg m^−2^ h^−1^ in 2020. The N_2_O fluxes were significantly and positively correlated with the soil water content and temperature during the growing seasons (Table S1).

Mulching (M), urea type (T), and year (Y) to the most extent affected the accumulated N_2_O emissions over the three growing seasons (Table 3). Regardless of fertilization, the cumulative N_2_O emissions under RM was decreased by 5.5% compared with NM. In addition, the cumulative N_2_O emissions were in the following order: C < CU < U. As desired, the cumulative N_2_O emissions was decreased by 14.0% and 5.9% under C and CU than U, respectively. However, no significant difference in cumulative N_2_O emissions was observed between U and CU under the same mulching method. Over the three growing seasons, RMC, RMCU, and NMC had significantly lower cumulative N_2_O emissions, with average reductions of 18.7%, 11.3%, and 14.3%, relative to NMU, respectively. Furthermore, RMC reached the minimum value of 891.7 g ha^−1^, which was 5.1–18.7% lower than those in the other treatments.

### Net global warming potential and carbon footprint

**Figure 3:**
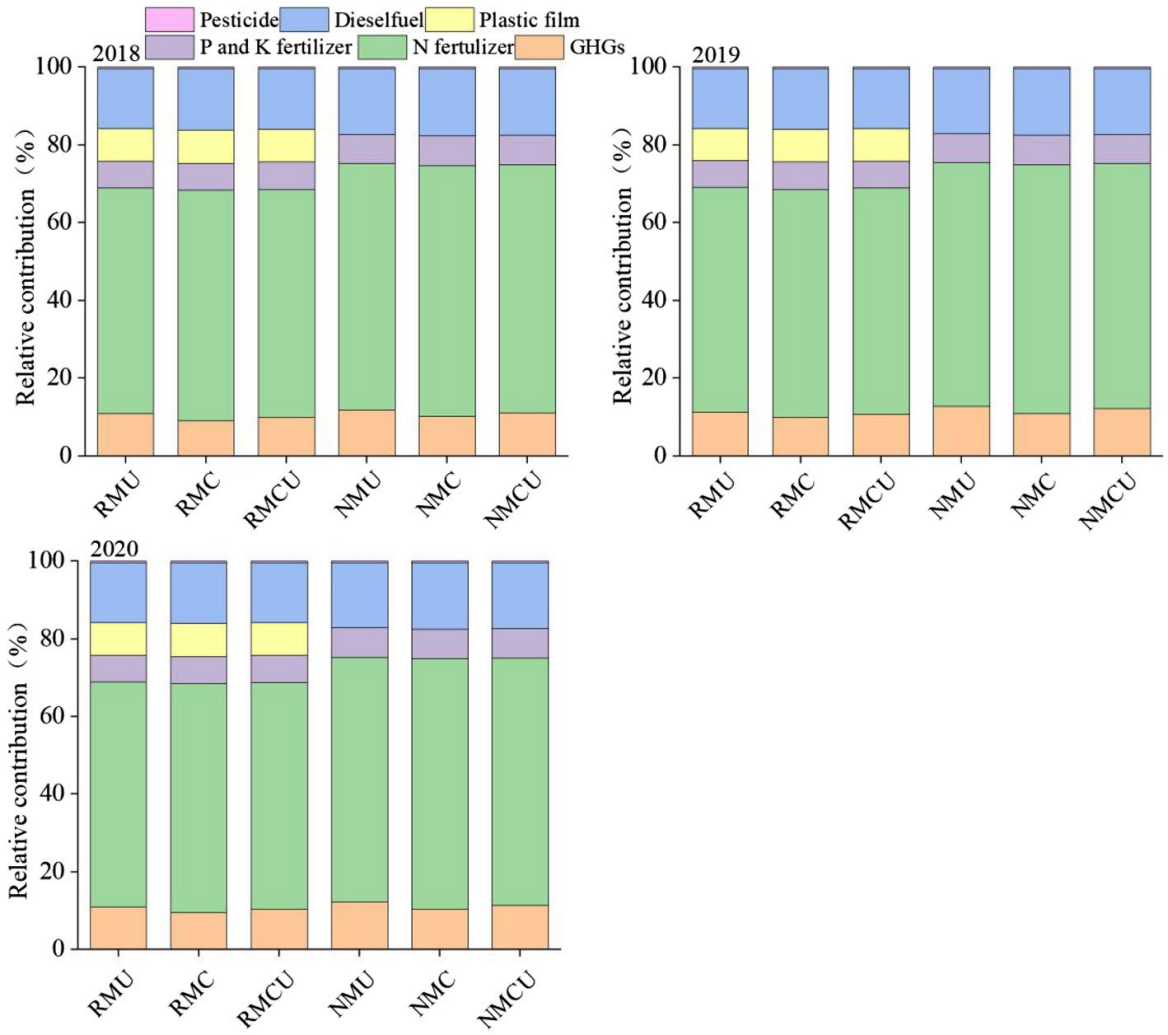
The relative contributions of different inputs to the total GHG emissions under different treatments in 2018–2020 growing seasons. *RM* plastic film mulching, *NM* no plastic film mulching, *U* conventional urea, *C* controlled-release urea, *CU* a mixture of equal amount of conventional urea and controlled-release urea at 1:1 ratio.

The relative contributions of components of NGWP for the potato growing seasons were compared across different treatments (Fig. 3). During the potato growing seasons, the production and transportation of N fertilizers was the largest contributor to NGWP, accounting for 61.1%. Emissions from diesel consumption by agricultural machinery operations from sowing to harvesting were the second largest component of NGWP, accounting for 16.3%. The GHGs were mainly dominated by N_2_O emissions across the three growing seasons, and the absorption of CH_4_ offset only a small portion of the NGWP, accounting for 10.8%. For RM (RMU, RMC, and RMCU) treatments, the fourth greatest contributor was plastic film, accounting for 8.4% of NGWP. In addition, applications of P and K fertilizers, and herbicides contributed only minimally to the NGWP of the potato cropping system. Mulching (M), urea type (T), and year (Y) significantly affected NGWP over the growing seasons (Table 3). Regardless of fertilization, NGWP was significantly higher under RM compared with NM (by 8.9%) across the three growing seasons. Irrespective of mulching, the order of magnitude of the NGWP for the three growing seasons was U > CU > C. Compared with U, averaged NGWP was significantly decreased under C by 1.9%, but there was no significant difference between U and CU. The RMU (2576.7 kg CO_2_-eq ha^−1^), RMC (2533.0 kg CO_2_-eq ha^−1^), and RMCU (2557.9 kg CO_2_-eq ha^−1^) showed a significant increase of 8.7%, 6.8% and 7.9% in NGWP compared with NMU (2370.7 kg CO_2_-eq ha^−1^), respectively. The lowest NGWP was obtained under NMC, on average 2322.8 kg CO_2_-eq ha^−1^ over the three growing seasons.

CF was also generally affected by mulching (M), urea type (T), year (Y), and M × Y (Table 3). The CF was 94.5–105.1 kg CO_2_-eq t^−1^ under RM, 101.3–109.5 kg CO_2_-eq t^−1^ under NM across the three growing seasons. Moreover, RM decreased CF by 2.8% under U, by 4.0% under C, and by 6.7% under CU respectively, compared to NM. On the other hand, on average, compared to C and U, the CU produced lower 8.8% and 7.4% of CF, respectively. RMCU and NMCU reached lower values, with decreases of 9.3–13.7% and 2.8–7.5% in CF compared with other treatments, respectively. On average, RMCU and NMCU significantly decreased CF by 11.8% and 5.5%, respectively, compared to NMU. CF under RMCU reached the minimum values of 76.8 kg CO_2_-eq t^−1^ in 2018, 112.7 kg CO_2_-eq t^−1^ in 2019, and 94.0 kg CO_2_-eq t^−1^ in 2020. The CF followed the order of 2019 > 2020 > 2018.

### Tuber yield and net ecosystem economic budget

Tuber yield was significantly influenced by mulching (M), urea type (T), and their interactions (Table 4). Compared with NM, the average tuber yield under RM increased by 9.4% in 2018, 17.2% in 2019, and 14.1% in 2020, respectively. Compared with U and C, the average tuber yield under CU significantly increased by 9.0% and 11.4% in 2018, by 5.8% and 10.7% in 2019, and by 7.5% and 10.6% in 2020, respectively. The tuber yield of RMCU and RMU was significantly higher than other treatments. Especially, tuber yield under RMCU reached the maximum of 33.2 t ha^–1^ in 2018, 22.8 t ha^–1^ in 2019, and 27.2 t ha^–1^ in 2020, which were respectively 12.5–23.3%, 6.8–30.7%, and 10.0–27.2% higher than other treatments. The tuber yield was the largest in 2018, followed by 2020 and 2019.

**Table 4:** Tuber yield and net ecosystem economic budget (NEEB) under different treatments in 2018–2020 growing seasons.

Year	Treatment	Yield (t ha^−1^)	Input (USD ha^−1^)	NGWP cost (USD ha^−1^)	NEBB (USD ha^−1^)
2018	RMU	29.5b	2581.7	41.8a	3857.0c
	RMC	29.1b	2674.1	41.0b	3672cd
	RMCU	33.2a	2627.9	41.4ab	4621.3a
	NMU	27.7c	2212.7	38.3c	3786.2c
	NMC	26.9c	2305.1	37.6d	3529.3d
	NMCU	29.2b	2258.9	37.9cd	4064.6b
2019	RMU	21.3b	2581.7	42a	2203.1b
	RMC	20.6b	2674.1	41.3b	1931.4c
	RMCU	22.8a	2627.9	41.7ab	2518.8a
	NMU	18.4cd	2212.7	38.7c	1811.7c
	NMC	17.4d	2305.1	37.9d	1480.9d
	NMCU	19.3c	2258.9	38.4c	1938.0bc
2020	RMU	24.7b	2581.7	41.8a	2622.7b
	RMC	24.2b	2674.1	41.1b	2370.0c
	RMCU	27.2a	2627.9	41.5ab	3092.4a
	NMU	22.2d	2212.7	38.5c	2402.9c
	NMC	21.4d	2305.1	37.7d	2150.0d
	NMCU	23.2c	2258.9	38.1cd	2592.0b
**Significance of factor**					
Mulching (M)		***	–	***	***
Urea type (T)		***	–	***	***
Year (Y)		***	–	**	***
M × T		**	–	NS	***
M × Y		NS	–	NS	*
T × Y		NS	–	NS	*
M × T × Y		NS	–	NS	NS

*RM* plastic film mulching, *NM* no plastic film mulching, *U* conventional urea, *C* controlled-release urea, *CU* a mixture of equal amount of conventional urea and controlled-release urea at 1:1 ratio.

Numbers followed by different letters in each column indicate significantly differences at α = 0.05 based on ANOVA test. The statistical significance is denoted by *P ≤ 0.05; **P ≤ 0.01; ***P ≤ 0.001, and *NS* not significant.

Mulching (M), urea type (T), year (Y) and their interactions had highly significant effects on NEEB, except for the M × T × Y (Table 4). The NEBB is an important reference indicator for crop production and agricultural activities. Over the three growing seasons, the mean NEBB varied from 2386.8 to 3410.9 USD ha^−1^. The highest NEEB were recorded under RMCU, while NMC consistently had the lowest NEEB. On average, the NEBB of RMCU was significantly increased by 17.8–42.9% compared with the other treatments. The NEBB followed the order of 2018 > 2020 > 2019. In addition, mulching (M), urea type (T), and year (Y) had highly significant effects on NGWP cost (Table 4). The RMCU, RMU, and RMC were more NGWP cost than other treatments. Compared with NMU, the NGWP cost increased by 7.9%, 8.7%, and 6.8% for RMCU, RMU, and RMC, respectively.

## Discussion

### Effect of mulching practices and urea types on soil CH_4_ uptake

Dryland soils normally are sinks for atmospheric CH_4_, owing to methanotrophs in soils oxidizing CH_4_ under dry conditions^46^. In our study, the CH_4_ uptake with small fluxes was observed in all treatments during the three growing seasons (Fig. 2a–c). This rate is almost consistent with that by Wang et al.^7^, who observed a CH_4_ sink with an average annual methane absorption (negative emission) of 940.8 ± 103.2 g CH_4_-C ha^−1^ year^−1^. Zhang et al.^47^ reported that soil hydrothermal properties were the main factors that affecting soil gas emissions. Nan et al.^16^ found that CH_4_ concentration was only weakly correlated with soil temperature and moisture because CH_4_ consumption was not sensitive to temperature. In the present study, the CH_4_ fluxes were significantly and positively correlated with the soil temperature, while a significant negative correlation was observed between CH_4_ fluxes and soil water content.

For rainfed potato fields, we found that cumulative CH_4_ uptake was decreased under RM in this study, in agreement with previous measurements^21,48^. This result is consistent with those of Yu et al.^17^, who found that the soil CH_4_ uptake was significantly lower under film mulching than under non-mulching in rainfed uplands. A possible reason for the lower CH_4_ uptakes in the RM was that the relatively low gaseous oxygen (O_2_) availability and the restricted CH_4_ gas exchange between the soils and the atmosphere under RM decreased CH_4_ oxidation by methanotrophs^19^. On the other hand, plastic film mulching can maintain higher soil water content and NH_4_^+^-N content, which can partially inhibit soil CH_4_ uptake^21^. In contrast, Chen et al.^49^ found that plastic film mulching increased CH_4_ absorption. The contradiction could be a result of the inconsistent investigations of agronomical measures, as well as the corresponding soil type, soil conditions, and meteorological characteristics. Agricultural practices, such as fertilization management and planting patterns, can affect the soil's ability to act as a sink of atmospheric CH_4_^50^. Much research has been conducted on the relationship between fertilization and CH_4_ emissions^50,51^. Zheng et al.^52^ found that the difference in CH_4_ flux between CU and U alone was also not significant over the two growing seasons. Our results show that the cumulative CH_4_ uptake did not vary among the three urea types, which is consistent with the report by Bai et al.^39^, due to the complex underlying mechanisms of methanogen activity.

### Effect of mulching practices and urea types on soil N_2_O emissions

In the present study, the flux of N_2_O emissions under different treatments peaked about 7–15 days after the base fertilizer applications, and then maintained relatively low levels due to the soil absorption and plant uptake of N, in line with the findings by Li et al.^53^. Fang et al.^41^ reported that the daily flux of N_2_O emissions under film mulching and N fertilization treatments peaked about 8 days post-sowing. The N_2_O is produced in soils essentially through the processes of nitrification and denitrification^54^, which were closely related to soil moisture and temperature^16^. In our study, significant and positive correlations were observed between N_2_O fluxes and soil water content and soil temperature during the growing seasons, indicating that improved soil moisture and temperature might become a more critical factor for soil N_2_O emissions, which is consistent with the previous studies^49,55^. It may be attributed to the fact that the high N_2_O fluxes following the precipitation events and higher temperature in this study (Fig. S2 and Fig. 2).

RM improves soil hydrothermal conditions, which probably facilitate N_2_O production through nitrification and/or denitrification processes^20^. In the present experiment, the RM had higher soil temperature and moisture contents, but RM had a negative effect on N_2_O emissions, supporting findings in previous studies^22,56^. Similar results also were found by Li et al.^21^, who reported that plastic film mulching enhanced soil temperature and water content but did not increase N_2_O fluxes. This result can be explained as follows. First, RM significantly reduced the soil mineral N content by increasing nitrogen use efficiency or the impervious barrier effects of the plastic film mulching on the gas exchange between soil and atmosphere, thereby offsetting N_2_O production^20,41^. Second, the lower N_2_O emissions in the RM could be attributed to increased soil denitrification by *Thiobacillus denitrificans*, which reduced some nitrous compounds further into nitrogen gas (N_2_)^21^. Thus, in the RM, N_2_O emissions are not only affected by soil temperature and moisture, but also may be related to soil nitrification and denitrification microorganisms and inorganic N content.

Increasing N_2_O emissions from agriculture are heavily linked with the application of mineral N fertilization^57^. It is very interesting that the soil N_2_O fluxes were consistent under U, C, and CU, but the use of C reduced soil N_2_O fluxes peaks. The application of C treatment significantly reduced cumulative N_2_O emissions relative to U by 14.0%. Similar results also were found by Zhang et al.^23^, who reported that the application of CRU instead of urea (same N rate) significantly decreased N_2_O emissions by 23.8%. These results may be explained that the N in uncoated urea is released more quickly than in CRU, thus the available N contents under U treatment are presumable higher than under C treatment. However, direct evidence cannot be provided. Future studies on the relationship between soil available N contents (NH_4_^+^-N and NO_3_^–^-N) and soil N_2_O fluxes need to be considered under various urea types. Moreover, the application of C, soil urease and the urea in the membrane were unable to directly contact, preventing the water transport required for urea dissolution in the membrane^58^. In addition, our results showed that the application of CU did not significantly reduce cumulative N_2_O emissions compared with U, this was consistent with the results observed by Bai et al.^39^.

### Effect of mulching practices and urea types on net global warming potential and carbon footprint

Different inputs of chemical fertilizers, human activities, and fuels create variations in carbon emissions from agricultural inputs under different management practices, indirectly influencing the carbon cycling of systems^59,60^. Many studies have reported that the net GWP is affected by the use of inorganic fertilizers, fuel, plastic film, and pesticides in the crop growing season^39,61^. Akhtar et al.^55^ reported that net GWP was significantly higher in the plots treated with straw mulch and N fertilizer. In our study, N fertilizer was the greatest contributor to the NGWP, and this result was consistent with the previous studies^62^. Therefore, optimizing N fertilizer application rates can be one of the key options to mitigate agricultural GHG emissions. In addition, diesel fuel also accounted for a large proportion of the NGWP. Our results showed that the total GHG emissions were significantly higher under RM than under NM, which was calculated by summating two GHG (N_2_O and CH_4_) fluxes and CO_2_ equivalent^19^. It should be kept in mind that each step involved in the use of plastic film, from manufacturing to its application under plastic film, leads to GHG emissions^63^. Our results showed that RM significantly increased the NGWP, which might counteract its positive effects, such as increases in crop productivity. This occurred mainly as a result of changes in plastic film, maybe because of the effect of indirect GHG emissions caused by the input of plastic film materials^64^ and lower soil CH_4_ uptake. Lee et al.^19^ reported that plastic film mulching highly increased NGWP via increasing GHG emissions. In addition, NGWP was decreased under C and CU compared with U. The decrease in NGWP might be related to the reduction of N_2_O emissions by the use of CRU.

In addition to a reduction in the NGWP, the main objective of this study was to maintain a low CF. CF based on the emission of CO_2_-eq per unit of crop production^63^, has been widely popularized and applied in the field of agriculture for the evaluation of environmentally friendly and clean production^65^. The factors influencing CF include the CO_2_ emissions from farmland soils and crops and indirect CO_2_ emissions from the production, storage, and transportation of agricultural production materials^1,66^. In our study, RMCU, RMU, and NMCU were very effective to reduce CF than other treatments, due to the high tuber yield increase rather than the NGWP increase. The present study suggested that RMCU had the lowest CF and was eco-friendly, which may be due to the effect of RMCU increasing the yield and could therefore offset environmental impacts to a greater degree. Bai et al.^39^ reported that the combination of urea and CRU in a one-time application significantly decreased the NGWP and CF of maize. Therefore, a better balance between production and environmental benefits can be achieved by RMCU in rainfed regions.

### Effect of mulching practices and urea types on tuber yield and net ecosystem economic budget

Plastic film mulching can improve grain yield by enhancing plant growth and nutrient absorption, especially in Northwest China^67^. In our study, the RMU, RMC, and RMCU increased soil water content (Fig. S2), hence effectively maintaining the high yield of potato (Table 4), which is consistent with the report by Tang et al.^32^. The key to a trade-off between yield, profit, and the environment in crop production is to achieve synchronization between N supply and crop demand to avoid N excess or deficiency. Guo et al.^10^ reported that CU was better synchronized with the N demand of rainfed crop relative to U and C, thus could accomplish a high grain yield. Our results illustrated that the RMCU had the maximum tuber yield over the three growing seasons. This result was mainly due to CU under RM can provide better water, temperature, and nitrogen conditions for crop growth to realize relatively high yield in semiarid farmland^32^. Additionally, in our study, tuber yield in 2018 was greater than that in the 2019 and 2020 growing seasons, which can be largely attributed to the higher precipitation in 2018 (Table 2).

In the present study, RM (RMU, RMC, and RMCU) treatment increased NGWP cost, which was mainly attributed to the significant increase in the NGWP. Under certain agricultural measures, NEEB represents the relationship between economic feasibility and environmental sustainability^28^. In this study, we found that RMCU, RMU, and NMCU were efficient ways to increase NEEB over the three growing seasons, due to high tuber yield would counterbalance the negative impact of increased input and ultimately increase the NEEB. This data shows that the RMCU can not only ensure high tuber yield but also reduce fertilizer costs, thereby achieving a high NEEB. Therefore, RM combination with CU (RMCU) could achieve the goal of high yield, NEEB, and low CF in rainfed regions.

Although our study indicated that RMCU practices have a lower carbon footprint and higher tuber yield and net ecosystem economic budget, the use of plastic film caused a large number of plastic film residues, which seriously pollute the environment (i.e., "white pollution" and "microplastic pollution"), damage soil structure, and hinder mechanical tillag^64^. Fortunately, biodegradable film or straw mulching has similar properties to plastic film, and could take the place of conventional plastic film to ensure cleaner agricultural production in the future. Future studies should also evaluate the effects of U and CRU at different mixing ratios, focusing on tuber yield, GHG emissions, and carbon footprint under ridge-furrow with plastic film mulching, considering the present study included only one mixed ratio of U and CRU.

## Conclusions

RM significantly decreased cumulative N_2_O emissions and CH_4_ uptake, but produced much higher NGWP when compared to NM. Among different urea types, the application of C was most significant in mitigating NGWP, which was mainly attributed to the reduced cumulative N_2_O emissions and increased CH_4_ uptake. However, the differences in cumulative N_2_O emissions, CH_4_ uptake, and NGWP were not significant between CU and U. In conclusion, the combined application of RM with CU (RMCU) not only helps achieve high yield and NEEB but also low CF for rainfed potato, which achieves a better balance between potato production and environmental benefits, was recommended as an efficient field management measure in dryland regions. Future studies should also evaluate the effects of U and CRU at different mixing ratios, focusing on tuber yield, GHG emissions, and carbon footprint under ridge-furrow with plastic film mulching. Furthermore, GHG emissions during fallow seasons may be measured and analyzed further to gain a comprehensive understanding of agricultural system emissions.

## Supplementary Information

Supplementary Information.
