# Supplementary material for: Blending controlled-release urea and urea under ridge-furrow with plastic film mulching improves yield while mitigating carbon footprint in rainfed potato
Source: Sci Rep. 2023 Mar 10;13:4018. doi: 10.1038/s41598-022-25845-4 (PMC10006086; doi:10.1038/s41598-022-25845-4)
Supplement: Supplementary file 1 — Supplementary Information. [file 41598_2022_25845_MOESM1_ESM.docx]

**Blending controlled-release urea and urea under** **ridge-furrow with plastic film mulching improves yield while mitigating carbon footprint in rainfed potato**


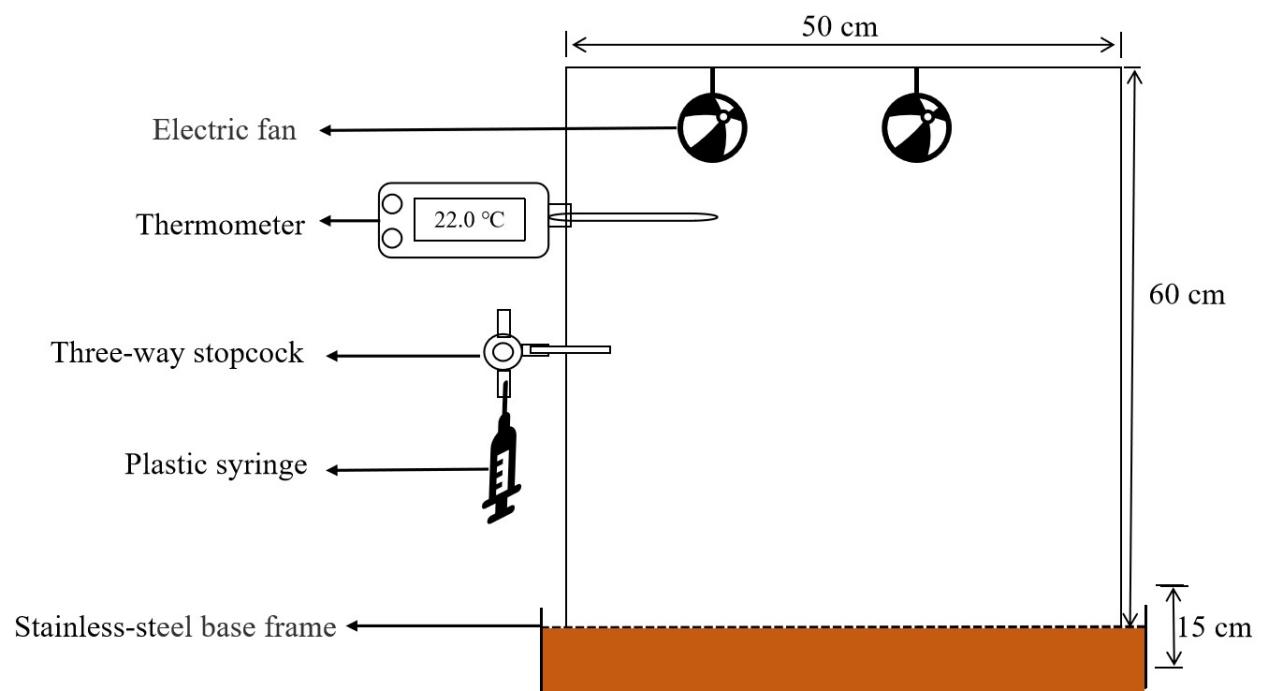


**Figure S1.** Diagram of the CH_4_ and N_2_O collecting device.

**
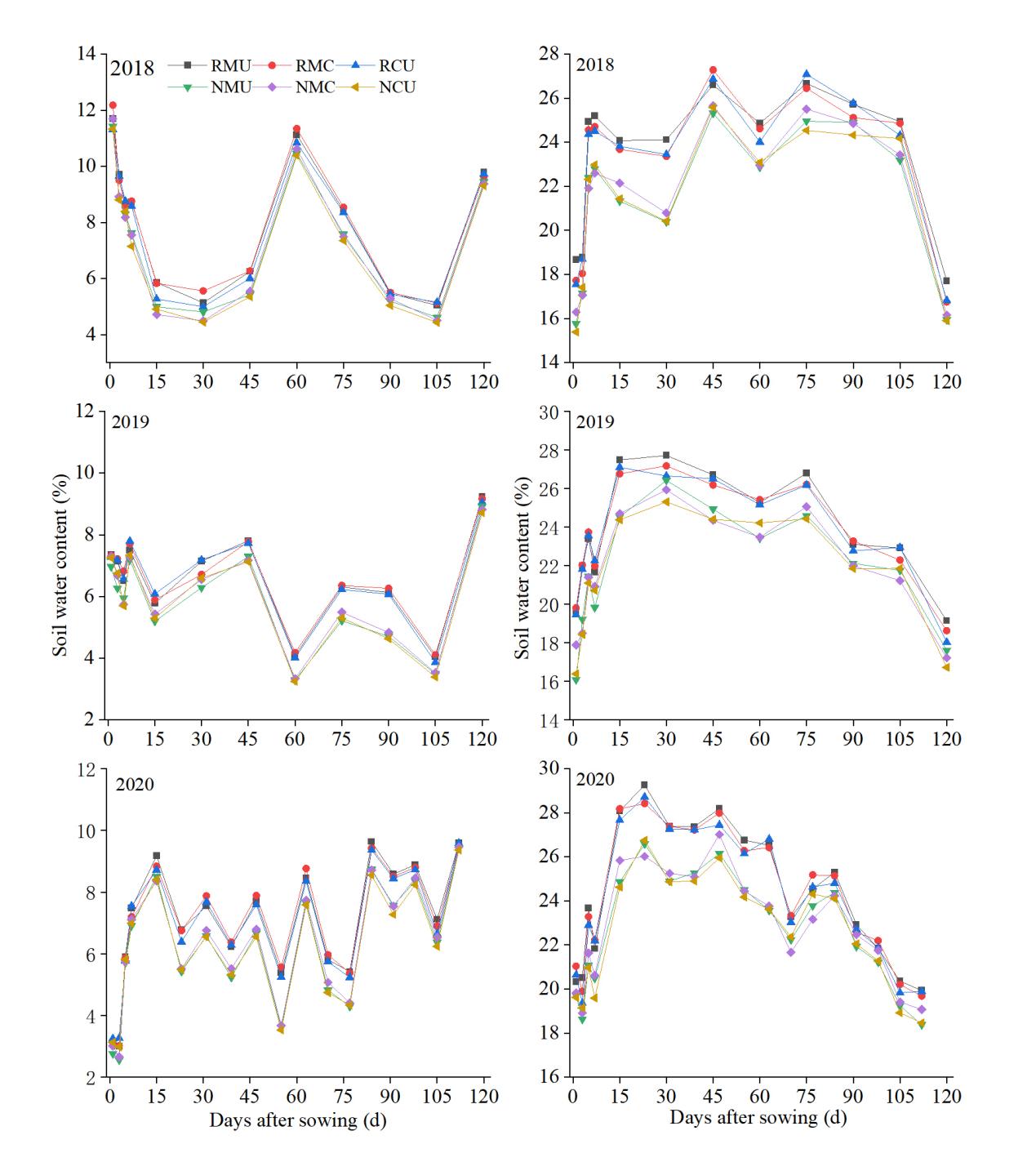
**

**Figure S2.** Soil temperature and water content at 0–20 cm depth under different treatments in 2018–2020 growing seasons.

Notes: RM, plastic film mulching; NM, no plastic film mulching; U, conventional urea; C, controlled-release urea; CU, a mixture of equal amount of conventional urea and controlled-release urea at 1:1 ratio.

**Table S1.** Pearson correlation of N_2_O or CH_4_ fluxes with soil water content or soil temperature for the potato growing season.

| Index | CH_4_ fluxes | | N_2_O fluxes | |
| --- | --- | --- | --- | --- |
|  | n | r | n | r |
| Soil water content | 252 | -0.25*** | 252 | 0.14* |
| Soil temperature | 252 | 0.14* | 252 | 0.35 *** |

Notes: n, number of observations; r, Pearson correlation coefficient; the statistical significance is denoted by *, P ≤ 0.05; **, P ≤ 0.01; ***, P ≤ 0.001.
